# A Case of Munchausen Syndrome Presenting with Hematemesis: A Case Report

**DOI:** 10.7759/cureus.1348

**Published:** 2017-06-13

**Authors:** Muhammad Aadil, Aniqa Faraz, Muhammad Jahanzaib Anwar, Maria Shoaib, Usama Nasir, Anum Akhlaq

**Affiliations:** 1 Department of Medicine, FMH College of Medicine and Dentistry; 2 Department of Internal Medicine, King Edward Medical University Lahore, Pakistan; 3 Department of Internal Medicine, Rush University Medical Center; 4 Department of Medicine, Dow Medical College, Karachi, Pakistan; 5 Medicine, C.M.H Medical and Dental College

**Keywords:** challenging diagnosis, factitious disorders, munchausen syndrome

## Abstract

Munchausen syndrome (MS) was first reported in 1951 by Richard Alan John Asher as a factitious disorder. It is a condition in which the patient intentionally produces symptoms to assume a sick role and gain medical attention. Underdiagnosis of this disorder results in the unnecessary use of medical resources, i.e. unnecessary medical tests and evaluations. This makes it one of the most challenging diagnosis in any medical set up. We present this rare case of a patient with chronic factitious disorder who presented to the emergency with hematemesis. The patient was successfully treated with psychotherapy with no recurrence. It is the need of the hour to create awareness amongst the medical and nonmedical population about factitious disorders so that they can easily be diagnosed and treated with efficiency. Further research is needed to find the risks leading to this syndrome and discover the cultural and social aspects of this disease, which may help us explore treatment strategies and avoid unnecessary tests and treatment.​

## Introduction

Munchausen syndrome (MS) was first reported in 1951 by Richard Alan John Asher in the Lancet. In this disease, patients intentionally produce physical or psychiatric illness symptoms to assume a sick role to gain medical attention [1]. Munchausen syndrome is a factitious disorder, which includes a group of conditions in which a patient falsely misinterprets, replicates or causes symptoms of injury or illness on themselves without any apparent external gains. This results in the unnecessary use of medical resources, i.e. unnecessary medical tests and evaluations, and this makes it one of the most challenging diagnosis in a medical set up. A recent study showed that the prevalence of factitious disorders is about 0.0001%–15%, though the exact prevalence of this condition in Pakistan is not known [2].

Here, we document this interesting and rare encounter of an elderly male from Pakistan, who presented to the emergency department with hematemesis and has been a diagnostic dilemma for a long time, till he was successfully diagnosed and treated as a case of Munchausen syndrome. This case report will contribute to raising awareness and understanding of the complex clinical presentations of Munchausen syndrome and how we can avoid overlooking such cases. This patient is currently being successfully treated with psychotherapy with no relapses.

## Case presentation

A 65-year-old man came to the psychiatric clinic with complaints of a lack of interest in pleasurable and daily activities, depressed mood, and multiple somatic complaints. He reported that he was doing fine two and half years back and that he first noticed these symptoms right after the death of a 23-year-old son in a road traffic accident. The intensity of these symptoms increased gradually with the passage of time and the occurrence of some financial problems.

His mood appeared depressed, and he explained that he feels “low” throughout the day. Crying spells and tearfulness accompanied it. Along with these symptoms, he also experienced some somatic and physical complaints like neck pain, headache, severe abdominal pain accompanied by vomiting, constipation, urgency and frequency of urination, shortness of breath, and marked loss of libido. A detailed physical examination was conducted, and relevant laboratory tests like the fecal occult blood test, ultrasound of the abdomen, and an electrocardiogram (ECG) were done. All of the test reports came back normal.

He strongly believed that he had diabetes mellitus and hypertension and explained that all of his symptoms are due to his diabetes mellitus. During illness, he had consulted multiple physicians asking them to treat him for diabetes mellitus, but none of the doctors diagnosed him with diabetes mellitus or hypertension.

Earlier in the year, he came to the emergency department with complaints of multiple episodes of hematemesis. He also complained of abdominal pain and mild headache. He reported that he had four episodes of blood-containing vomitus. All relevant investigations like complete blood count (CBC), basic metabolic panel, liver function test, serum amylase and lipase, thyroid stimulating hormone (TSH), and T4 levels were ordered again. Endoscopy was ordered too. The patient reported that since his last visit he also consulted few other psychiatrists in the outpatient department and started taking over-the-counter sedatives for his disturbed sleep. On further questioning, he had no history of obsessions, head injury, seizures, mania, substance abuse, or suicidal attempt. He explained that his illness had a marked impact on his social and occupational aspects of life and that he was becoming socially isolated. The patient was then admitted to the hospital ward.

His past medical and psychiatric history did not reveal anything significant. His physical examination, mini mental state examination, and baseline investigation came out within reference range again.

In the ward, he appeared more comfortable. He avoided talking about what triggered his vomiting or other somatic complaints but discussed his past life in great detail. During the stay, he had no episode of hematemesis, abdominal pain or constipation. After discussing the case with his other primary doctor, a diagnosis of factitious disorder was made. He was counseled in an empathetic manner, and a family member was contacted who then got involved in his care as well. The patient started getting psychotherapy treatment and was discharged from the hospital after one week. He was followed up at an outpatient service with no new complaints.

This patient had made multiple visits to hospitals, clinics, and the emergency department (ER) for the last two years. He claimed to have chronic diseases like diabetes and hypertension and also had an acute presentation to the ER with a complaint of hematemesis, though there was no evidence of bleeding in the presence of any family member or in the hospital setting. His recurrent negative clinical and lab evaluations lead to the diagnosis of Munchausen Syndrome.

## Discussion

A factitious disorder may present with physical or psychological symptoms intentionally produced by the patient with no visible gains. The features that distinguish Munchausen from other types of factitious disorders include presentations that are more alarming and refractory with worse outcomes. Many patients with Munchausen syndrome present with complaints of bleeding and as it is an alarming symptom, it mostly leads to extensive investigations and hospitalization [3-4]. To diagnose a case of factitious disorder, the Diagnostic and Statistical Manual of Mental Disorders, Fifth Edition (DSM-5) lists the following criteria [5]:

a) Making up physical or psychological signs or symptoms or causing injury or disease with the deliberate intention to deceive b) Pretending to be sick or injured or to be having problems functioning c) Continuing with the deception, even without receiving any visible benefit or reward d) Behavior is not better explained by another mental disorder, such as a delusional disorder or another psychotic disorder

The typical characteristics that should prompt the physician to include Munchausen syndrome in the diagnosis include deliberately lying, repeatedly coming to the clinic/hospital with similar complaints in a short span of time, taking excessive drugs (especially insulin and warfarin) to induce side-effects, recurrent abdominal pain, scars on limbs, and rheumatologic and hematological disorders. The lack of electronic medical records in Pakistan makes it easier for such patients to fake history and laboratory findings, and that is why a high index of suspicion is required. In a case report by Koufagued K, a patient presented with self-injection of the needle in a limb, which resulted in subcutaneous emphysema. In the emergency room, this patient was aggressive and did not provide any explanation for the needle in her limb and arm swelling. When her previous record was checked, there was a history of multiple such presentations to four different hospitals in the past. This is the reason why such cases are usually diagnosed late and are subject to unnecessary investigations as the patient pretends to have similar symptoms as those with the actual disease. Most doctors are not suspicious and focus on the acute presentation and the likely management. Such patients are subjected to unnecessary medical interventions and hospitalizations, which can be more detrimental in the long run [3].

A brief literature review showed that there can be several risk factors predisposing to this disease including serious illness during childhood, loss of a loved one, personality disorder, and being a healthcare worker. Our patient’s mood changes, somatic and physical complaints started gradually after he lost his young son in a road traffic accident, a likely cause of the onset of this syndrome. These patients are unable to control their mimicking behavior, and the motive is mostly to gain sympathy and attention [6].

The most important factors affecting the prognosis of these patients are early recognition of disease, psychiatric referral, and the presence of depression or personality disorder [7]. The management of Munchausen syndrome is also very challenging and includes a great deal of tolerance on the part of the physician and requires a strengthened patient-therapist alliance to develop the patient's conscious self control to minimize the feigned illness symptoms [8]. Reports of the successful treatment of such syndromes are limited and many emphasize the importance of the therapist-physician alliance. A tolerant attitude towards the patient is important to establish a strong relationship. We also recommend that the primary care physicians collaborate with mental health providers after establishing a strong physician-patient relation to provide a better health care support system for such patients suffering from factitious disorders, specially in a culture where mental health disorders are still stigmatized. The mental health provider can then focus on either therapeutic or medical treatment depending upon the underlying psychiatric condition, for example, mood disorder, anxiety disorder or borderline personality disorder. After the diagnosis of our patient, treatment of his underlying depression, regular outpatient visits, and psychotherapy sessions resulted in an excellent prognosis for our patient.

We recommend that physicians all across the globe should report more cases of Munchausen syndrome. More research is required in this arena to understand the cultural, social, and psychological aspects of Munchausen syndrome and to find out which treatment strategy can be most beneficial for such patients. This will help us develop a culturally sensitive treatment approach.

## Conclusions

Munchausen syndrome is a diagnostic dilemma that needs to be given adequate medical and social attention by encouraging further research and spreading awareness not only amongst the general population but also health care providers. With proper evaluation, diagnosis, and psychotherapy, the disease will not remain a diagnostic dilemma and would be easier to control and treat. This case report will contribute towards the awareness of physicians about Munchausen syndrome and the strategies to diagnose and treat it.
